# Supplementary material for: Medication adherence and its impact on glycemic control in type 2 diabetes mellitus patients with comorbidity: A multicenter cross-sectional study in Northwest Ethiopia
Source: PLoS One. 2022 Sep 21;17(9):e0274971. doi: 10.1371/journal.pone.0274971 (PMC9491880; doi:10.1371/journal.pone.0274971)
Supplement: Attachment — Submitted filename: Comments to authors.doc [file pone.0274971.s002.doc]

**Comments to authors**

Thank you for an opportunity to review this article. This study aims to identify factors related to medical adherence in patients with type 2 diabetes in Ethiopia. I have some comments as following;

**Introduction:**

The authors provide the prevalence of diabetes worldwide and in the Ethiopia. However, the introduction should be concise.

**Methodology:**

Regarding sampling, it is unclear the number of participants from each hospital is unequal. More explanation is required. Did the laboratory test was extracted from medical chart? Who was in charge for data collection?

Regarding the study setting, it might be a good idea to add a short introduction of the study setting to readers at the first part of the methods section,

In outcome measures, it was redundant in explanation of the GMAS instrument. I would rather move GMAS paragraph to outcome measure part. And write; Medication adherence was measured by using GMAS…… to measure how ……….. I think no need for operational definition in the article because it should be included in the instrument explanation. Have the authors asked for permission in using the GMAS instrument?

**Results:** No comments

**Discussion:**

The novelty of the findings relative to prior literature should be identified. All factors are common in the medical adherence among diabetes patients and have been published. Discussion should be provided in the context of Ethiopia; why these factors related to medical adherence among Ethiopia population and the study settings.
