# Supplementary material for: Medication adherence and its impact on glycemic control in type 2 diabetes mellitus patients with comorbidity: A multicenter cross-sectional study in Northwest Ethiopia
Source: PLoS One. 2022 Sep 21;17(9):e0274971. doi: 10.1371/journal.pone.0274971 (PMC9491880; doi:10.1371/journal.pone.0274971)
Supplement: Attachment — Submitted filename: Response to Reviewers.docx [file pone.0274971.s004.docx]

**Responses to the review’s comments**

**Dear PlOS ONE Academic Editor,**

Thank you for giving us the opportunity to submit a revised draft of the manuscript and we would also like to thank your constructive and fruitful comments and suggestions on our paper **(**Manuscript ID: **PONE-D-22-20901R1**). We are very concerned and combined all the suggested comments provided, which we believe that strengthened the paper and we hope this render our paper to be considered for publication in your reputed journal. We appreciate the time and effort that you and the reviewers dedicated to providing feedback on our manuscript and are grateful for the insightful comments on and valuable improvements to this paper.

We authors would like to let you know that all comments and concerns raised by both academic editors and reviewers are fully addressed and indicated with track changes in the main document and a point-by-point response letter for the editors and reviewers.

**Comments from the editor:**

**1#.... Journal requirements:**

Please review your reference list to ensure that it is complete and correct. If you have cited papers that have been retracted, please include the rationale for doing so in the manuscript text, or remove these references and replace them with relevant current references. Any changes to the reference list should be mentioned in the rebuttal letter that accompanies your revised manuscript. If you need to cite a retracted article, indicate the article’s retracted status in the References list and also include a citation and full reference for the retraction notice.

**Author reply*:*** Thank you for your comments and recommendations to ensure that the reference is complete and correct.

We have checked the whole references using Google scholar searching. Consequently, we have found some errors on reference 13. Accordingly, we had made it correct and we also include the DOI and indicate the change with track changes. In addition, when we have searched reference number 32 on Google scholar, we have not found it and then we have looked the original article on google searching. Now we ensured it was correctly cited. We also include the DOI. Other than these two references, we have not found any error on our best searching, all listed references are found on Google scholar. However, in case any retracted reference is still included, it is not doing purposely and it might be unknowingly. If so, it would be my pleasure if you indicate me which one is retracted. Thank you in advance for your hard work and positive comments.

**Additional Editor Comments:**

**2#.......**Based on the reviewer's opinions, I agreed with the reviewer 1 that some minor issues need to be revised before accepted to publish.

**Author response*:*** We authors are very thankful for your concerns and suggestions. We had revised the raised issues and the changes are tracked. The manuscript changes have tracked and all the raised issues, changes and responses are indicated with those line numbers in the manuscript with tracked changes. We hope that you have found it has improved.

**Response to Reviewers’ comments:**

**Reviewer #1**

I would like to thank the authors for their consideration of my comments and working on it accordingly. However, I would suggest that some of the parts detected in the comments were not properly addressed. Specifically, I would like from the authors to address the following suggestions:

**#1...** Page 3/ Lines 26-29: The sentence included the word "patients" three times which is not needed. Also, the end of the sentence to write "are factors that influence medication adherence ..."

**Author response*:*** We authors are very thankful for your deep concerns and suggestions. We have accepted the recommendations and made editing and corrections for the whole parts you mentioned. The manuscript changes have tracked and all the raised issues, changes and responses are indicated with those line numbers in the manuscript with tracked changes. We hope that you have found that it has improved. **Page 3/ Lines 26-29.**

**#2....** Page 3/ Line 29, Page 4/ lines 1-3: I would suggest choosing the single percentage or the range of percentages if I am allowed to.

**Author reply*:*** Thank you very much. It was to implicate rate of medication nonadherence to patients with comorbidity and patients with T2DM, respectively. But based on your recommendation and it was already mentioned earlier, we had revised and choose the one with ranges of percentages. **Page 3/lines 29 and page 4/lines 1-2.**

**#3**…Page 5/ Lines 6-8: You could start the sentence with: to be included in the study, participants should be adults (aged 18 years or older), diagnosed with diabetes, and are diagnosed with at least one comorbidity.

**Author reply*:*** Thank you very much for your positive comments for improving the quality of paper. Base on your kind recommendation, we had revised the sentence. Page **5/lines 5-6.**

**#4....** Page 4/ line 6, Page 11/ Line 7: the sentence "enhance negative consequences" does not make sense. From my point of view, you cannot enhance negative things. Therefore, I would suggest changing to possibly "increase the risk for negative consequences."

**Author reply*:*** Thank you for your positive comments. We made it correct and revised accordingly, **page 4/line 4 and page 11/line 5.**

Generally, we the authors are grateful for the comments provided to improve the quality of the paper. We also have a great appreciation for your efforts and assistance for improving the paper. Thank you in advance.

**Reviewer #2**

Authors has rewritten all issues according to suggestion. This study is very interesting and will add some valuable knowledge in the diabetes study.

**Author reply*:*** We the authors are grateful for the comments provided to improve the quality of the paper. We also have a great appreciation for your efforts and assistance for improving the paper. Thank you in advance.
